# Spermatozoa: A Historical Perspective 

**DOI:** 10.22074/ijfs.2018.5316

**Published:** 2018-06-20

**Authors:** Jenniffer Puerta Suárez, Stefan S. du Plessis, Walter D. Cardona Maya

**Affiliations:** 1 Reproduction Group, Department of Microbiology and Parasitology, Medical School, University of Antioquia, Antioquia, Colombia; 2 Division of Medical Physiology, Faculty of Medicine and Health Sciences, Stellenbosch University, Tygerberg, South Africa

**Keywords:** Fertility, History, Male Reproductive Physiology, Sperm

## Abstract

The 100,000^th^ scientific article on the subject of spermatozoa was recently published. Numerous studies evaluated the characteristics of this important cell that led to tremendous discoveries. Since its first observation and description in 1677, many important characteristics have been described regarding this highly fascinating gamete. In this review, we intend to provide a historical account of the numerous milestones and breakthroughs achieved related to sperma- tozoa. We conducted a review of the literature by selecting the most important subjects with regards to spermatozoa. Since their discovery by van Leeuwenhoek, spermatozoa have been studied by scientists to better understand their physiology and process of interaction with their female counterpart, the oocyte, in order to treat and resolve infertility problems. Three centuries after van Leeuwenhoek’s discovery, the 100,000^th^ article about these cells was published. It is encouraging that sperm research reached this landmark, but at the same time it is clear that further research on male reproductive physiology and spermatozoa is required to shed more light on their function and pathology in order to reduce the number of unexplained infertility cases.

## Introduction

Few cells have attracted the world’s attention and intrigued scientists throughout history as much as the male gamete or spermatozoon. It is one of the most fascinating and important cells. A crucial part is involved in the fecundation process due to its role in the delivery of male genetic material and proteins to the oocyte at the time of fertilization. Despite the vital dependence of human life on the interaction between gametes, research on human and male reproduction is less than infectious diseases. This is perhaps due to the emergent and expansive nature of infections. Only 0.4% of all scientific papers published over the last 50 years have pertained to spermatozoa despite their discovery in 1677 by van Leeuwenhoek (1). van Leeuwenhoek did not act alone; his observations were made in the company of his assistant, Ham, which demonstrated the importance of scientific cooperation and communication.

In 2016, the 100,000^th^ paper related to spermatozoa was published. To celebrate this significant milestone, we aim to highlight and discuss the most important events related to the history of this cell that travels from one individual to another in order to initiate and preserve life. We conducted an extensive evaluation of PubMed [National Center for Biotechnology Information (NCBI)] literature using the term “sperm” without any limitation on the date of publication. In addition, we determined the numbers of publications on another topic of major interest to the scientific community-viral infection caused by the human immunodeficiency virus (HIV). Furthermore, we evaluated the relationship between HIV and sperm articles in the last 30 years, and conducted a review of the literature about the most important findings. We selected and discussed milestones that pertained to spermatozoa.

###  Historical review

 In the database used for this analysis (PubMed), the number of results retrieved with the term “sperm” was much greater than when we used the term “spermatozoa”. The 100,000^th^ article on HIV was published in the year 2000 (3,102,842 articles had been published when last accessed on January 8th, 2017, Fig.1). This milestone was already achieved 16 years before the 100,000^th^ publication on spermatozoa (101,787 articles had been published when last accessed on January 8^th^, 2017).

During June 1843, according to PubMed, there were three manuscripts published. Two papers, one original and one editorial (2, 3), dealt with the presence of sperm in the hydrocele from three patients. The third paper was a letter that concerned sperm from a dromedary (4). In contrast, the first paper about HIV was published nearly 140 years later in September 1982. This publication by the center for disease control (CDC) analyzed the reports received that pertained patients with the acquired immune deficiency syndrome (AIDS) (5).

**Fig.1:**
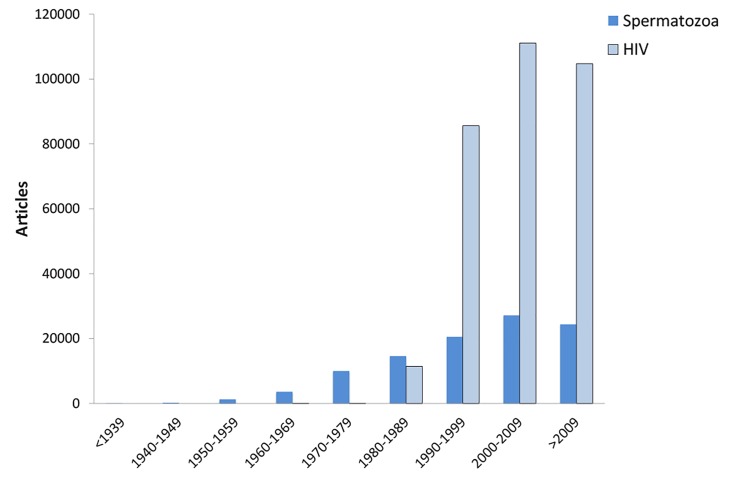
Number of articles published in relation to time on human immunodeficiency virus and spermatozoa.

It is important to note that since 1990 the relationship between articles published about HIV and sperm were similar (median 4.2-fold more for HIV, range: 3.4 to 4.8). In addition, there were significantly more (P<0.0001) numbers of papers published during the last three decades (1990-2016) in PubMed about HIV (median 10.4, range: 5.8 to 15.7) compared to spermatozoa (median 2.6, range: 1.7 to 3.6, Fig.1).

 Quantity does not necessarily imply quality. However, good quality manuscripts have been published about sperm and HIV. We intend to discuss a few of these publications to highlight some of the events that have marked the history of research on human reproduction (Table 1) from the perspective of the male gamete -the spermatozoon.

Important events related to spermatozoa include the advent of assisted reproduction, male contraception, and the effects of the interaction of sperm with microorganisms (viruses, bacteria, and fungi), among others. However, the story of the discovery of sperm has originated more than three centuries ago, in 1677. Without any scientific purpose and purely driven by human curiosity, van Leeuwenhoek and his assistant described animalculae (6) in human semen. It was not until 1776 that interest in the "new male gamete" began to focus on its physiology. Spallanzani noted that it became motionless when cooled by snow. Additionally, Spallanzani performed the world’s first artificial insemination of a viviparous animal. He artificially and successfully inseminated a bitch, which led to the birth of a live puppy by using spermatozoa obtained directly from the reproductive tract of a dog that had died (7).

It is impossible to discuss sperm without referring to the environment in which it develops. Cooper (8) published a book “observations on the structure and diseases of the testis” in which he described the place of origin of sperm, i.e. the testicle. In the first chapter he cited: “The testes are contained within the scrotum, in which they are suspended at unequal heights, the left testis generally hanging lower than the right.” The manuscript was not only limited to a description of this organ, but the author also recounted the anatomy, physiology, and alteration in the male reproductive system in general.

The development of spermatogenesis is connected to the testicular anatomy and the action of mainly two cell types: Leydig cells, first described by the German histologist Leydig in 1850 (9), and Sertoli cells, originally described by the Italian histologist Sertoli in 1865 (10). Leydig cells are the main source of testosterone, which is essential for spermatogenesis and sexual differentiation. One of the main targets for testosterone is the Sertoli cell that surrounds and nourishes germ cells during spermatogenesis.

The spermatozoon’s physiology, as well as any alterations to it that could lead to infertility, prompted the development of techniques used to evaluate some important characteristics of sperm during the reproductive process. In 1866, Sims devoted a chapter of his book “Clinical Notes on Uterine Surgery” to describe the nature and properties of semen, artificial fertilization, and the conception period. Sims wrote: “If we take a drop of semen from the vagina immediately after sexual intercourse and place it under the microscope, we shall see the hurried movements of seemingly thousands of spermatozoa”; thus was born the post-coital test to “measure” the capability of sperm to penetrate a woman’s cervical mucus. Huhner has described the details of this technique where it is known as the Sims-Huhner test (11).

Oocyte-sperm interaction and the interaction of sperm with the environment have been studied extensively from two perspectives: agglutination and chemotaxis. These phenomena were particularly studied in marine species such as sea urchins. Simple experiments combined with detailed observation allowed Lillie (12) to describe the production of sperm isoagglutinin by the ova of two marine species, *Arbacia punctulata* and *Nereis aibuhitensis* as early as 1912. Lillie’s findings have shown that oocytes produce a substance which causes sperm agglutination for 3 to 5 minutes. The agglutination is totally reversible. However, it does not imply a return to the original physiological state, as the movement of spermatozoa gradually cease. After 10 minutes they no longer have the capability of fertilization. The researcher has concluded that sperm penetration is due to biochemical reactions and not solely mechanical properties of the cell. This biochemical response has been subsequently termed chemotaxis, a type of guidance system for spermatozoa to the oocyte. Sperm guidance may involve more than one chemoattractant and include thermotaxis, which is based on slight temperature differences. These guidance processes rely on the precise timing of Ca^2+^ transients that control flagellar beating and the swimming trajectory. An asymmetric waveform is associated with turns or bends in the trajectory, whereas more symmetric beating results in a straighter swimming path. Both the oocyte and cumulus cells secrete chemoattractants. A number of these molecules have been implicated in capacitation, hyperactivation, and eliciting the acrosome reaction (13).

Spermatozoa are highly specialized cells with specific energy requirements. Adenosine triphosphate (ATP), which constitutes the basis for supporting the key functions of the spermatozoa, is formed through the glycolysis and oxidative phosphorylation pathways. To date, a discrepancy has been reported as to which method of ATP production is primarily utilized by the spermatozoa for successful fertilization (14). McCarthy et al. (15) conducted one of the studies on sperm metabolism that explored the presence of glycolysis in semen in 1928. They reported that the sugar concentration in semen from 50 men decreased by 70 to 90% after one day of incubation.

In 1929, Macomber and Sanders (16) were the first to publish a paper on a reference value for sperm count based on the results from nearly 300 men. They showed that although pregnancy was possible with less than 60 million sperm/ml, the probability increased with sperm counts. During the same period, Carey and Hotchkiss, Mason, and Moench and Holt (17) reported the importance of sperm morphology assessment during evaluation of infertility cases based on their findings that correlated this parameter with pregnancy outcome. They also reported the initial reference values for morphology. They stipulated that: i. The number of abnormal sperm heads must not exceed 19 to 20% in a normal semen sample, ii. Impaired fertility could be assumed if sperm head abnormalities reach 20 to 23%, and iii. When the sperm head abnormalities were above 25%, clinical sterility usually prevailed.

In 1932, Baskin (18) described the first contraceptive vaccine to be directed against spermatozoa. In this study the author inoculated women with sperm and triggered the production of anti-sperm antibodies, which resulted in infertility. Only one of the 20 women in his study became pregnant. Of great significance was the fact that the immunization lasted for about one year and that the process appeared to be harmless for women. However, the most important criticism for this study was directed towards the antibodies produced, as these proved to be non-specific because they had the ability to bind to spermatozoa used for vaccination as well as to other individuals, and could react with other proteins in somatic cells.

 Another important milestone was reached in 1942, when Lasley et al. (19) developed a staining technique to differentiate between live and dead sperm based on the fact that live ram sperm were impermeable to several stains.

The first suggestion that oxidative stress might play a role in the etiology of defective sperm function came from MacLeod, one of the pioneers of modern andrology. As early as 1943, MacLeod demonstrated that human spermatozoa rapidly lost their motility in oxygenated medium via mechanisms that could be recovered by the concomitant presence of catalase, a specific scavenger of hydrogen peroxide (reactive oxygen species) (20).

Oxidative stress is one of the major causes of defective sperm function and male infertility because it causes lipid peroxidation and disrupts the integrity of sperm DNA. This, in turn, can affect sperm motility and limit fertilizing potential as a result of collateral damage to proteins and lipids in the sperm plasma membrane. In addition, reactive oxygen species increase as a consequence of leukocyte infiltration. A variety of primary factors can initiate oxidative stress such as infection, age, obesity, and exposure to a variety of adverse environmental influences. Despite the resultant oxidative damage to the sperm’s chromatin, once fertilization occurs, the oocyte can repair most of the damaged sperm DNA (21).

In 1947, Dan (22) explored the enigma of sperm entrance into the egg and discovered the acrosome reaction, “an event of exocytosis by which spermatozoa expose the devices essential for penetration through the egg coats and for fusion with the egg plasma membrane, and is species-specific and triggered by signals from the eggs or their appendices”. Dan employed a very primitive model of electron microscopy to study and describe this phenomenon. His work demonstrated that many groundbreaking discoveries were based on careful observation of specific natural phenomena and not purely on the use of advanced technologies. Although the use of new technologies has allowed us to analyze problems in greater detail and from a different perspective, the best instrument for scientific research remains human curiosity and acute observation.

After the initial discovery of sperm and identifying the relationship of sperm with fertility, investigations started to focus more intensively on the process of sperm transfer to the oocyte. During the first in vivo fertilization trials in rabbits, it was observed that the sperm must remain in the fallopian tubes for six hours prior to ovulation, thereby pointing to a possible physiological change that the spermatozoa have to undergo in the female tract (23).

Spermatogenesis occurs in the testis, after which the sperm move to the epididymis for final maturation. Once ejaculated, sperm can undergo two phenomena, capacitation and the acrosome reaction, which thereby renders them able to fertilize oocytes (24). The metabolism of epididymal spermatozoa differs from that of ejaculated spermatozoa, thus underpinning the influence of the environment on their physiology. During the natural sequence of mammalian fertilization, the oocyte awaits the arrival of spermatozoa in the fallopian tubes after their relatively rapid transit from the vagina to the upper part of the tube. This time interval is for the spermatozoa to acquire fertilization capacity and not allow accumulation of a large quantity of spermatozoa, since the number of sperm present at the site of fertilization is relatively small (23). Once the first sperm penetrates the oocyte, its cortical granules undergo exocytosis and release their content into the perivitelline space. This leads to the modification of the zona pellucida and subsequently the blockage of polyspermy. The second polar body is formed after the second mitotic division (25).

In the middle of the 20^th^ century, researchers analyzed sperm structure to deduce phylogenetic relationships between taxa. Variations in the size, shape, and physiology of spermatozoa in the animal kingdom and their relation to the method of reproduction captivated the biologists of the time. Between 1940 and 1950, with the development of the electron microscope, ultra-thin dissection, and new staining techniques, researchers could observe the structure of spermatozoa from various species. One of the most interesting findings was made by Franzén (26), in 1955 and 1956. He suggested a causal relationship between sperm morphology and the mode of fertilization of the species. In 1950, Leuchtenberger and Schrader (27) reported that the acrosome contained the lytic enzyme hyaluronidase.

Soon after, it was suggested that sperm morphology related to pregnancy. Macleod and Gold (28) were the first to propose that a possible relationship existed between sperm motility and fertility. They performed a study on 2000 semen samples and reported that the average percentage of active cells was higher in the fertile group of proven fathers (n=1000) than in the group of infertile men (n=1000).

 Spermatozoa undergo selection during their travel along the female reproductive tract. Different selection techniques have been developed *in vitro* to identify and choose sperm of higher quality (29-31). In 1958, Bhattacharya (29) reported that it was possible to separate rabbit sperm using colloid medium and centrifugation (gradient centrifugation method). The author claimed to be able to discriminate between X- and Y-bearing sperm with this technique. At a later stage (1971), Drevius (30) used bull semen to show that motile spermatozoa could swim upwards following centrifugation (swim-up method). When incubated for a specific period of time, only motile sperm were present in the supernatant. Another popular method used to increase the percentage of motile sperm was proposed by Tea et al. (31) in 1984 (migration-gravity sedimentation methodology), who used semen samples from normo- and asthenozoospermic individuals. During semen incubation in the Jondet tube, two built-in concentric tubes, and based on the migration ability of sperm and the sedimentation phenomena, motile human spermatozoa jumped into the central tube.

Amann and Katz (32), the pioneer of computer aided sperm analysis (CASA), in 1978 presented the first system to track movement. Initially, these systems were extremely expensive and the results were not reproducible. Presently, a number of different CASA systems have become available for sperm motion detection. CASA made the comprehension and assessment of various motility and kinematic parameters possible with great reproducibility and complete objectivity. However, the validity of the results is conditioned by the proper use of the CASA system. Recommendations include: i. The user of the system must be highly trained, especially in sample preparation, ii. Internal quality and external controls should be included, iii. The results should be compared directly with manual assessments, iv. The depth and temperature of the chamber should not affect sperm motility, and v. A permanent record of samples should be kept if reanalysis is required (33).

Yanagimachi et al. (34) developed the sperm penetration assay by using zona pellucida-free oocytes from hamsters. The authors showed that freshly ejaculated human spermatozoa were incapable of binding and penetrating zona-free hamster ova. However, after 4 or more hours of incubation, the sperm increased their ability to interact and penetrate these oocytes.

In 1979, a letter to the editor was published in The Lancet that informed the world about the birth of Louise Brown on July 25^th^, 1978. Her mother had a 9-year history of infertility. This pregnancy was achieved after laparoscopic recovery of an oocyte, IVF, and reimplantation of an embryo which was incubated for 2.5 days under laboratory conditions. This important achievement by Steptoe and Edwards (35) resulted in the first baby born through IVF.

In 1980, Rothman (36) has reported, for the first time, the postmortem retrieval of sperm. This procedure is important when the family requests sperm preservation after death to honor the wish of fatherhood by the deceased.

Bleil and Wassarman (37) examined the structure and function of the zona pellucida and subsequently described it in 1980 as a “relatively thick, translucent, acellular coat which surrounds the plasma membrane of fully grown mammalian oocytes”. It was not until 1990 that they discovered the ZP3 protein, a sperm receptor localized on the surface of the egg’s zona pellucida that serves as sperm receptor and acrosome reaction-inducer. They concluded that the sp56 sperm protein binds to ZP3 during adhesion of the gametes that precedes fertilization.

 In 1980, Evenson et al. (38) reported an alternative method to assess semen quality. They have described the sperm chromatin structure assay (SCSA) as a highly useful test to determine the validity of male breeding by comparing the sperm chromatin of fertile and infertile men based on similar results previously observed in bulls. The basic semen analysis evaluates sperm count, motility and morphology, but there are numerous cases where these parameters are all within the “normal” range, yet the men are infertile. The SCSA test uses acridine orange dye, which emits a red fluorescence when it binds to single-stranded DNA and a green fluorescence when it binds to double-stranded DNA. The relationship between both types of fluorescence evaluates DNA heterogeneity and fragmentation, and can possibly account for the infertility of men who have a normal semen analysis (39). During the last decade, this test has been an important tool for the diagnosis of fertility due to the relation between a sucSpermatozoa cessful pregnancy and DNA status.

During the previous century, it became generally accepted that assessment of the basic seminal parameters was the cornerstone for evaluation of male fertility. For this reason, the World Health Organization (WHO) published its first manual on the examination of human semen (WHO, 1980). Since then, the manual has been regularly updated with new versions published in 1987, 1992, and 1999; each edition has contained new proposals, the latest changes, and revised cut-off values for the semen variables. After rigorous scrutiny, the latest edition was published in 2010. This edition is believed to be one of the most comprehensive manuals to date because the authors have based updated semen variables on real life data which included men of proven fertility and secondly presented results as lower reference limits (5^th^ percentile) (40).

In 1981, Yanagimachi (41) noticed that spermatozoa began to move in an extremely vigorous swimming pattern just prior to initiation of the acrosome reaction. This process was termed hyperactivation. The human sperm capacitation and acrosome reaction could be induced *in vitro* by the addition of albumin, which mediated cholesterol efflux and hyperactivation. This unique movement pattern is considered to give spermatozoa the strong thrusting power that allows them to penetrate the zona pellucida, and occurs on completion of the capacitation process (42).

The hypo-osmotic swelling test (HOS) was developed in 1984 to evaluate the functional integrity of the human sperm membrane. The principle of this technique is based on the fact that human spermatozoa “swell” under hypo-osmotic conditions due to the influx of water and subsequent membrane expansion. This technique is extremely important because membrane integrity is vital for sperm metabolism and the correct change in membrane properties required for a successful union with the female gamete. HOS can thus be used in the diagnosis of male infertility. Investigators have stated that any compounds that change osmolality and significantly enhance sperm swelling should be removed from IVF media (43).

Later, Kruger et al. (44) described the specific morphological characteristics of human spermatozoa in 1988. They stated that the following criteria should be met for a spermatozoon to be regarded as normal: the head (length: 5 to 6 μm and width: 2.5 to 3.5 μm) has to be oval with a well-defined acrosome (approximately 40 to 70% of the sperm head) and there should be no neck, midpiece, or tail defects. In addition, no cytoplasmic droplets should more than half the size of the sperm head. They concluded that morphologic features played a significant role in the probability of pregnancy.

In 1988, Hodgen et al. (45) published an editorial entitled “The Hemizona Assay (HZA): Finding Sperm that have the “Right Stuff’ ”. In this editorial they reported the story of two infertile men who sought a new diagnostic method for their problem. To quote, they “needed quantifiable objective assessments of sperm quality having a high reliability, a low cost of operation, ease of application, a wide availability, and rapid results”. In brief, we wished for a male-factor "litmus test”. With the advent of IVF, they have ushered in the first direct laboratory assessment of a man’s fertilization potential through the HZA. Tight binding of human spermatozoa to the human zona pellucida is an early critical event in gamete interaction, which is needed for fertilization and activation of development. In reality, this test only assesses the ability of sperm to interact with the egg *in vitro*. However, this binding step is believed to provide unique information that predicts the sperm’s ultimate fertilizing potential (46). Briefly, a viable oocyte is micro-dissected into two equal parts. Subsequently, each matching half is exposed to the same concentration of sperm from the patient (test) and a proven father (control). The semen samples are prepared using swim-up separation to obtain motile sperm and co-incubation lasts 4 hours. The hemizonae are removed and technicians count the number of sperm tightly bound to the outer surface. The HZA index is calculated by a simple equation: number of test sperm bound divided by the number of control sperm bound ×100 (45).

The ability of sperm to act as vectors for foreign DNA was first published in 1971 by Brackett et al. (46) who used rabbit sperm and simian virus 40. However, it was only in 1989 when Lavitrano et al. (47) published the first sperm-mediated gene transfer in mice using a pSV2CAT plasmid that this methodology became more popular. Since then, successful *in vitro* uptake of exogenous DNA, including viral DNA, by sperm of different animal species has been reported.

In 1992, the landscape of the assisted reproduction technique (ART) was transformed with the first reports of intracytoplasmic sperm injection (ICSI). This technique allows the direct injection of a single spermatozoon into the ooplasm of metaphase-II oocytes, after which the embryos are placed in utero.

 In the same year of ICSI development, Carlsen et al. (48) warned about a genuine decline in semen quality over a period of 50 years. They described a decrease in mean sperm count from 113×10^6^/ml in 1940 to 66×10^6^/ml in 1990, and in seminal volume from 3.40 ml to 2.75 ml among men without a history of infertility. This finding could be related to a concomitant increase in the incidence of genitourinary abnormalities, such as testicular cancer, and possibly cryptorchidism and hypospadias, which impact male gonadal function. The analysis was based on a total of 61 papers published between 1938 and 1990 that included data on 14947 men. Some researchers criticized the ‘Carlsen study’ based on the study design, the heterogeneity of the groups investigated, statistical evaluation, non-standardized methodology used for semen analysis, and variation in abstinence times. The authors of one specific manuscript who opposed this spermatic crisis article concluded with a sentence to motivate future research: “it must be acknowledged that the research group that led us down the wrong path also demonstrated how we can get back on the proper research track” (49).

 Sperm-specific ion channels or CatSper has been described in 2001 by Ren et al. (50). CatSper is a six-transmembrane-spanning repeat of the voltage-dependent Ca^2+^ channels located in the principal piece of the sperm tail. The sperm from CatSper knockout mice showed poor motility and could not fertilize eggs with an intact zona pellucida. CatSper is considered as a target for the design of a possible contraceptive. The evaluation of the functionality of CatSper has also been considered useful in the detection of male infertility.

 The identification of proteins in sperm was based principally on the use of anti-sperm antibodies, isolation and identification of individual protein spots from 2D gels and on infertility models in the mouse which involve mutations that affect sperm capacitation and motility. For example, to identify critical sperm-oocyte fusion antigens such as the IZUMO protein, Inoue et al. (51) used anti-sperm antibodies.

 As already demonstrated, sperm physiology has been studied in humans as well as in various animal models that have contributed substantially to this scientific field. For example, the first systematic molecular study of sperm composition that utilized Drosophila melanogaster sperm samples was performed by Dorus et al. (52) and represented the first characterization of the eukaryotic cell content.

Additionally, in 2006, a novel gamete receptor known as beta 1,4-galactosyltransferase 1 (GalT) that mediates sperm adhesion to the zona pellucida, was described (53). Such key receptors considered vital for the fertilization process are not only important for infertility, but also for the development of new male contraceptive targets. The mechanisms that mediate sperm-egg binding in the mouse involve a GalT-ZP3-independent mechanism, which mediates initial sperm-egg adhesion followed by a GalT-ZP3-dependent interaction that contributes to acrosomal exocytosis.

One of the most rewarding moments in the history of sperm and sperm research occurred when Edwards was awarded the 2010 Nobel Prize in Physiology or Medicine for the development of IVF. In his biography, Edwards was described as competitive “all determined to win or, if not to win, to go down fighting”. During his childhood, he developed an enduring curiosity about agricultural and natural history, in particular the reproductive patterns of some animals. This curiosity, through multiple experiments such as sperm labeling led to evaluation of the kinetics of spermatogenesis *in vivo* (54).

Spermatogenesis is one of the most complex and longest processes of sequential cell proliferation and differentiation known to man. Therefore, the *in vitro* production of functional spermatozoa has long been pursued. In 2011, the first successful attempt was reported. The authors produced spermatids and sperm by *in vitro* culturing and maintained spermatogenesis for more than 2 months. They subsequently performed round spermatid injection (ROSI) and obtained 12 offspring. These animals were proven fertile as examined by brother-sister mating. The researchers therefore demonstrated that the organ culture conditions, without a circulatory system as *in vivo*, could support complete spermatogenesis of mice. This finding will definitely contribute to the development of new diagnostic and therapeutic techniques for male infertility (55).

**Table 1:** Milestones in the history of spermatozoa


Year	Author	Milestone	Reference

1677	Howards	Sperm cell discovery	(6)
1776	Mann and Lutwak-Mann	Spermatozoa temporarily immobilized by cooling	(7)
1830	Cooper	Observations on the structure and diseases of the testis	(8)
1850	Leydig	Leydig cells	(9)
1866	Huhner	Post-coital test	(11)
1888	Ebner	Sertoli cells	(56)
1878	Dudley et al.	Spermine	(57)
1912	Lillie and Kaupp et al.	The production of sperm iso-agglutinine by ova	(12, 13)
1929	Macomber and Sanders	The spermatozoa count	(16)
1932	Baskin	Temporary sterilization by injection of human spermatozoa	(18)
1940	Charny	First testicular biopsy	(58)
1942	Lasley et al.	Staining method for sperm viability	(19)
1943	MacLeod	The first suggestion that oxidative stress might play a role in the etiology of defective sperm function	(20)
1948	Pegg	Cryopreservation	(59)
1950	Dan	Acrosome reaction	(22)
1951	MacLeod and Gold	First seminal parameters proposed-fertility standard	(28)
1951	Chang and Austin	Capacitation	(23, 25)
1951	Tulloch	Varicocele as a cause of infertility	(60)
1953	Bunge and Sherman	Living child after insemination with semen from a sperm bank	(61)
1953	Kleegman	Donor insemination	(62)
1958	Bhattacharya	Density gradient	(29)
1969	Baccetti and Afzelius	The First International Symposium on Spermatology	(63)
1971	Drevius	Swim-up	(30)
1978	Amann and Katz	Computer aided sperm analysis (CASA)	(32)
1976	Greep and Chang	Distinguished andrologist award	(64)
1976	Yanagimachi et al.	Sperm penetration assay (SPA)	(34)
1978	Steptoe and Edwards	Development of *in vitro* fertilization (IVF)	(35)
1980-2010	World Health Organization (WHO)	Range of arbitrary threshold values for normal human semen	(40)
1980	Bleil and Wassarman	Structure and function of the zona pellucida	(37)
1980	Evenson et al.	Sperm chromatin structure assay (SCSA)	(38, 39)
1980	Rothman	Postmortem sperm retrieval	(36)
1981	Yanagimachi	Hyperactivation	(41, 42)
1984	Jeyendran et al.	Hyposmotic swelling test (HOS)	(43)
1984	Tea et al.	Migration-gravity sedimentation method	(31)
1988	Kruger et al.	Sperm morphologic features	(44)
1988	Hodgen et al.	Hemizona assay (HZA)	(45)
1989	Lavitrano et al.	Methods for sperm-mediated gene transfer	(65)
1990	Bleil and Wassarman	ZP3-binding protein	(37)
1992	Palermo et al.	First intracytoplasmic sperm injection (ICSI)	(66)
1992	Carlsen et al.	Evidence for decreasing quality of semen during past 50 years.	(48)
1994	Ogura et al.	Birth of normal young after electrofusion of mouse oocytes with round spermatids	(67)
1995	Wilcox et al.	Probability of conception	(68)
1996	Stief et al.	Sildenafil	(69)
2001	Ren et al.	Sperm-specific ion channel - CatSper	(50)
2006	Shur	Identification of novel gamete receptors - beta1,4-galactosyltransferase-I	(70)
2010	Johnson	Nobel Prize for development of IVF	(54)
2011	Sato et al.	*In vitro* production of functional sperm	(55)


## Conclusion

In this journey through the history of sperm, we have observed how human curiosity prompted the description of natural phenomena and the attempts made to try to explain these phenomena. Strictly speaking, it is impossible to discover facts of nature that have been in existence for thousands of years. We therefore clarify and define the use of the word ‘discover’ as "looking with new eyes", which encompasses acute observation, critical thinking, relating ideas, and questioning those events that we encounter on a daily basis as part of the miracle of life. Advances in modern technology allow us to find answers to rephrased Pleaquestions and analyse them from a new perspective in our attempt to explore and understand complex functional and molecular processes.

Physiological knowledge of each human organ system and cell allows us to understand not only how the human body works, but also where and how alterations, specifically in the male reproductive tract, may affect the continuation of life. Understanding sperm biology allows for a significant impact on infertility. For example, by increasing the chance of fertilizing the oocyte with enhancing the male gamete’s presence at the site of fertilization. New technologies allow us to investigate unexplained male infertility and possibly lead to the discovery of new and novel treatment solutions, thereby reducing the prevalence of cases of idiopathic infertility.

Finally, this review reminds us that the greatest advantage of recollecting history is that it can solve old problems with new methodologies that understand their nature. We can conclude that despite numerous advances and new insights published in these 100,000 articles, more research on human reproduction, especially on spermatozoon, are needed to help reduce the overall numbers of patients who suffer from infertility.

As authors of this review and seeing the historical development of this magnificent cell, we thank the researchers who are part of this journey in knowledge for their incredible contributions and invite new researchers to remember that curiosity is the best human attribute. We apologize to authors whose important contributions we could not cite due to limitations in reference number.
